# Violence Against Women Who Inject Drugs

**DOI:** 10.1001/jamanetworkopen.2026.2096

**Published:** 2026-03-17

**Authors:** Samantha Colledge-Frisby, Shelly Walker, Anna Lee Wilkinson, Bek Petrovic, Lisa Maher, Daisy Gibbs, Gail Gilchrist, Sophia Schroeder, Amanda Roxburgh, Marika Burgess, Peter Higgs, Sione Crawford, Nadia Gavin, Paul Agius, Bernadette Ward, Joseph Doyle, Nico Clark, Mark Stoové, Paul Dietze, Ashleigh Cara Stewart

**Affiliations:** 1 National Drug Research Institute, Curtin University, Melbourne, Australia; 2 Disease Elimination, Burnet Institute, Melbourne, Australia; 3 School of Public Health and Preventive Medicine, Monash University, Melbourne, Australia; 4 School of Population and Global Health, University of Melbourne, Melbourne, Australia; 5 Kirby Institute, University of New South Wales, Sydney, Australia; 6 National Addiction Centre, King’s College London, London, United Kingdom; 7 Harm Reduction Victoria, Melbourne, Victoria, Australia; 8 Faculty of Health, Deakin University, Burwood, Victoria, Australia; 9 School of Rural Health, Monash University, Bendigo, Australia

## Abstract

**Importance:**

Women who inject drugs disproportionately report experiencing violence, yet they are overlooked in interventions and policy responses.

**Objective:**

To quantify and contextualize experiences of violence among Australian women who inject drugs.

**Design, Setting, and Participants:**

This mixed-methods cohort study used data from SuperMIX, a longitudinal cohort of people who inject drugs. Self-reported survey data from January 1, 2008, to December 31, 2019, were linked with administrative health records (hospital, emergency department, ambulance, and mortality). Qualitative interviews with 10 women between October 1 and November 30, 2023, explored experiences of violence and service access. Women were recruited via convenience sampling carried out across Melbourne, Australia, in areas with known drug markets and needle and syringe programs. Statistical analysis was performed between May and August 2025.

**Main Outcomes and Measures:**

Experience of assault or violence.

**Results:**

A total of 431 women were included, with a median age at baseline of 29 years (IQR, 25-34 years). By 2019, combining self-report and administrative data, 353 women (82% [95% CI, 78%-86%]) had experienced at least 1 assault, and 164 (38% [95% CI, 36%-43%]) had experienced at least 1 sexual assault. In administrative data, 82 of the 431 women (19% [95% CI, 15%-23%]) had ever had an assault-related hospital admission, 101 (23% [95% CI, 19%-27%]) had an assault-related emergency department presentation, and 74 (17% [95% CI, 14%-21%]) had an assault-related ambulance attendance. A total of 304 women (71%) completed at least 1 annual follow-up survey (total, 1677 follow-up surveys), of whom 178 (59% [95% CI, 53%-64%]) reported an assault since their last interview. Among 409 follow-up surveys reporting assault, seeking health care was reported in approximately one-third (128 [31%]). Qualitative findings revealed barriers to seeking health care, including stigma, fear of child protection involvement, lack of trust in clinicians, and logistical challenges.

**Conclusions and Relevance:**

In this mixed-methods cohort study of Australian women who inject drugs, violence against women was pervasive and severe, yet rates of seeking health care remained low likely due to intersecting structural and social barriers. Recognition of the burden of violence is a critical first step in ensuring tailored responses to violence that meet the needs of marginalized women.

## Key Points

**Question:**

What is the burden of violence and assault among Australian women who inject drugs?

**Findings:**

In this mixed-methods cohort study of women who inject drugs, survey data (2008-2019) were linked with administrative health records, and qualitative interviews were conducted. A total of 431 women were included, of whom 82% experienced at least 1 assault and 38% experienced at least 1 sexual assault.

**Meaning:**

This study suggests that violence is pervasive among women who inject drugs in Australia, underscoring the need to prioritize them in violence prevention and response efforts.

## Teaser

This cohort study uses survey data linked with administrative health records and qualitative interviews to quantify and contextualize experiences of violence and assault among Australian women who inject drugs.

## Introduction

Violence against women results in considerable harms.^1^ Women who use drugs are at increased risk of violence, with a lifetime prevalence of experiencing violence estimated at 70% to 98%.^2,3,4,5^ Drug use and violence against women are complex and nonlinear; drug consumption can precede violence, result from exposure to violence, and/or stem from structural disadvantage that increases the risk of violence.^6,7,8^ However, evidence shows that women who have experienced violence are more likely than women who have not to use alcohol and other drugs,^8^ and women who use alcohol and other drugs are more likely than women who do not to report experiencing violence.^7^

Poor outcomes from violence are amplified for women who inject drugs.^9^ Among women in Spain who injected drugs, nearly half reported physical or sexual assault in the previous year; these women were more likely to be unhoused, engaged in sex work, and report a previous sexually transmitted infection.^10^ Among Black women in New York City using illicit drugs, each increase in violence (eg, physical, sexual, or emotional) experienced was associated with a 27% increased risk of drug overdose.^11^ A review found intimate partner violence was associated with substance use and impeded treatment engagement and completion.^8^ Managing these harms is complicated by barriers to support, as stigma, fear of child protection involvement, and discriminatory service exclusion often deter women who use drugs from seeking help after violence.^12^

In October 2022, the Australian government released the National Plan to End Violence Against Women and Children.^13^ Although the 10-year plan recognized the increased risk and burden among Aboriginal and Torres Strait Islander women, culturally and linguistically diverse women, LGBTQIA+ (lesbian, gay, bisexual, transgender, queer, intersex, asexual, and other sexual and gender minority) communities, and women with disabilities—both in terms of experiencing violence and being misidentified as perpetrators of violence—it does not acknowledge the unique burden and vulnerabilities faced by women who use or inject drugs. This may reflect a lack of contemporary evidence. In response, we examined data from a prospective cohort study with linked administrative health records and qualitative data to document experiences of violence among women who inject drugs in Melbourne, Australia.

## Methods

### Study Design and Setting

This mixed-methods cohort study used a convergent design,^14^ drawing on findings from 2 studies analyzed separately and brought together to enhance interpretive insight. Data were sourced from SuperMIX, a community-recruited cohort of people who inject drugs in Melbourne, Australia (established 2008).^15^ Convenience sampling was conducted across Melbourne (ie, metropolitan Melbourne, Frankston, Dandenong, and Geelong) in locations with known drug markets, through needle and syringe programs, and via street outreach. Researchers administered hour-long annual surveys using an electronic data capture tool (REDCap)^16^; participants were remunerated for participating (AUD$40 in 2019).^17^ Participants who provided written consent were linked with statewide health and mortality records^15^ and invited to participate in qualitative substudies. This study adheres to the Strengthening the Reporting of Observational Studies in Epidemiology (STROBE) reporting guideline and statement for reporting cohort studies and the American Association for Public Opinion Research (AAPOR) reporting guideline for survey research. Ethical approval was obtained from Human Research Ethics Committees at the Victorian Department of Health and Human Services, Victorian Department of Health, and the Alfred Hospital.

The quantitative component of the study used longitudinal survey and administrative health (ambulance, emergency department [ED], hospital, and mortality) data collected from January 1, 2008, to December 31, 2019. The qualitative component was designed to complement and contextualize quantitative findings by exploring women’s experiences of sexual and reproductive health care and access to violence-related services.

### Participants

SuperMIX participants were aged 18 years or older, injected drugs monthly for the previous 6 months, and provided informed consent and the details required for data linkage (sex, date of birth, and health care number). Participants who self-reported their gender as female or woman at baseline were included in the qualitative analysis.

Between October 1 and November 30, 2023, we conducted a qualitative substudy of the sexual and reproductive health care needs of women who use drugs. Ten cisgender women from SuperMIX were purposively recruited based on recent drug use, with those known to the research team approached directly due to the study’s sensitivity. One woman declined to participate. Hour-long semistructured interviews included questions on accessing violence-related services and participants received AUD$50. These findings were used to contextualize and interpret patterns identified in the quantitative data, rather than as a standalone qualitative study.

### Data Sources

#### Self-Report

Data from baseline and follow-up surveys conducted from 2008 to 2019 included sociodemographic characteristics, social circumstances, drug use, physical and mental health, access to health and social services, and experiences of violence. Items on violence determined whether women had been assaulted, how many times, and by whom (eTable 1 in Supplement 1). Women were also asked if and where they sought medical attention after experiencing violence, with options including a general practitioner (GP), the ED, primary health care center, counselling, or elsewhere. These questions were asked at baseline (lifetime recall) and at each follow-up interview, covering the period since the previous interview (approximately 12 months).

#### Administrative Data

The Victorian Admitted Episodes Dataset included statewide public hospital admissions from 2008 to 2019. We used *International Statistical Classification of Diseases, 10th Revision, Australian Modification* (*ICD-10-AM*) diagnosis codes (≤40 per presentation) to determine physical or sexual assault (*ICD-10-AM* codes X85-Y05 and Y07-09), sexual assault only (Y05), and physical assault only (X85-Y04 and Y07-09).

The Victorian Emergency Minimum Dataset detailed statewide ED presentations from 2008 to 2019. We used *ICD-10-AM* coding outlined to identify assault-related presentations. We considered the human intent variable—the clinician-assessed likely role of human intent in external injury. All events coded as “sexual assault by anyone”; “neglect, maltreatment, assault by anyone”; “child neglect, maltreatment by parent, guardian”; or “assault not otherwise specified” were considered assaults.

The Victorian Ambulance Clinical Information System captured statewide ambulance data from 2008 to 2019. We used ambulance-specific event coding to determine callouts for assault (eTable 2 in Supplement 1).

The National Death Index captured deaths up to December 31, 2019, including date of death as well as primary and additional causes of death. We used the *ICD-10-AM* coding outlined to identify assault-related deaths.

### Statistical Analysis

Statistical analysis was performed between May and August 2025. We calculated the number and proportion (95% CI) of women reporting lifetime and recent assaults at baseline and follow-up, then combined these data to derive cumulative self-reported assaults by December 31, 2019, categorized as any assault, sexual assault, and physical assault. Administrative data (2008-2019) were analyzed separately for assault-related hospital admissions, ED presentations, ambulance attendances, and deaths. Cumulative counts and proportions were derived by adding women with self-reported assault to women with assault recorded only in administrative data (eTable 2 in Supplement 1). Combined baseline and follow-up self-report data were used to characterize perpetrators, assault types, and health care accessed.

To describe health care seeking, we conducted a subanalysis of women who self-reported assault in at least 1 follow-up survey and summarized sociodemographic and assault characteristics overall and by seeking health care (no or yes). Survey data included age at interview (years); Aboriginal or Torres Strait Islander status (yes or no); housing stability (stable, unstable [excluding sleeping rough], and sleeping rough); paid work (yes or no); income (no income, $1-$599/week, or $600-$2500/week); sexuality (heterosexual or nonheterosexual), child removal (lifetime and past 12-month history); living arrangements (alone with partner, alone with children, with children and partner, or another arrangement [eg, with friends, family, or housemates]); injecting drugs alone (<50% or ≥50% of the time), paid sex work (yes or no); injecting frequency (daily or more than once a day, or less than daily); recent imprisonment (yes or no); GP access in past 12 months (yes or no); and current opioid agonist treatment (yes or no).

To identify characteristics associated with health care seeking after assault, we fitted a generalized linear population-average binomial model for the dependent variable of seeking health care, using complete-case data. Covariates included self-reported sexual and physical assault and all other relevant covariates listed, selected based on evidence as modifiable barriers and enablers to seeking health care after assault.^11,18,19^ Variables with substantial missingness were excluded to avoid excessive loss of observations. Time was modeled continuously to reflect each person’s sequence of follow-up surveys and baseline health care seeking (eFigure in Supplement 1). Results are presented as odds ratios (ORs), unadjusted and adjusted (AOR), with 95% CI and *P* values derived from Wald tests (2-sided), with *P* < .05 considered statistically significant. Analyses were conducted in Stata, version 18 (StataCorp LLC).^20^

To describe experiences of accessing services for violence, qualitative interviews were audio-recorded, transcribed verbatim, pseudonymized, and managed in NVivo software, version 15 (Lumivero).^21^ Using a deductive codes framework based on the interview guide and study aims, all transcripts were coded and then refined inductively through comparison and constant review, with codes collapsed into higher-level categories. A second-level analysis focused specifically on women’s experiences of violence and service access, further refining codes to develop thematic insights for the mixed-methods analysis.

## Results

Of 1485 SuperMIX participants from 2008 to 2019, 431 women were included. The median age at baseline was 29 years (IQR, 25-34 years), 67 (16%) identified as Aboriginal and/or Torres Strait Islander, the median age at injecting initiation was 17 years (IQR, 15-21 years), 31 (7%) were unhoused, and 55 (13%) were living in unstable accommodation at baseline. The 10 women in the qualitative substudy had a mean age of 47 years (range, 38-60 years).

### Women Who Experienced Assault, Characteristics of Assault, and Health Care Seeking

Two-thirds of women (297 [69%; 95% CI, 65%-73%]) reported at least 1 assault, and one-third (144 [33%; 95% CI, 29%-38%]) reported sexual assault at baseline (Table 1). A total of 304 women (71%) completed at least 1 follow-up survey (1677 surveys), of whom 178 (59% [95% CI, 53%-64%]) reported at least 1 assault since their last interview. In administrative data, 82 of 431 women (19% [95% CI, 15%-23%]) had a hospital admission with an assault diagnosis, including 10 with sexual assault. Of 431 women, 101 (23% [95% CI, 19%-27%]) had an assault-related ED presentation, and 74 (17% [95% CI, 14%-21%]) had an assault-related ambulance attendance. Combining self-report and administrative data up to 2019 showed most women (353 of 431 [82%; 95% CI, 78%-86%]) had been assaulted, and 164 of 431 women (38% [95% CI, 36%-43%]) had been sexually assaulted. Among women who self-reported assault (n = 345), most (181 [53%]) identified their partner as the assailant, while 149 (43%) reported being assaulted by a stranger (Table 2).

**Table 1.** Self-Reported Experiences of Violence and Records of Assault-Related Health Care Episodes Among Women in the SuperMIX Cohort, Australia, 2008-2019

Self-report data	No. (N = 431)	% (95% CI)
History of assault at baseline (N = 431 at baseline)		
Any type of assault	297	69 (65-73)
Sexual assault	144	33 (29-38)
Physical assault	273	63 (59-68)
Assaulted since last interview (ie, study follow-up) (n = 304 women who had a follow-up)^a^		
Any type of assault	178	59 (53-64)
Sexual assault	39	13 (9-17)
Physical assault	167	55 (49-61)
Lifetime history of assault^b^		
Any type of assault	345	80 (76-84)
Sexual assault	168	39 (34-44)
Physical assault	326	76 (72-80)
Administrative data, 2008-2019 (N = 431)		
Assault diagnosis, hospital	82	19 (15-23)
Sexual assault diagnosis, hospital	10	2 (1-4)
Assault-related presentation, ED	101	23 (19-27)
Sexual assault diagnosis, ED	0	0
Assault-related ambulance call-out	74	17 (14-21)
Assault-related mortality	0	0
Combined self-report (baseline and follow-up) and administrative data (N = 431)		
Any assault		
Self-report baseline	297	NA
Self-report follow-up only (not in baseline)	35	NA
Administrative data (not in self-report)	21	NA
Total any assault^c^	353	82 (78-86)
Sexual assault		
Self-report baseline	144	NA
Self-report follow-up only (not in baseline)	16	NA
Administrative data (not in self-report)	4	NA
Total any sexual assault^c^	164	38 (36-43)

Abbreviations: ED, emergency department; NA, not applicable.

^a^ Prevalence is defined using women who completed a follow-up interview as the denominator (n = 304).

^b^ Self-report at either baseline or at follow-up interviews.

^c^ Experienced (sexual) assault according to self-report and health administrative data.

**Table 2.** Self-Reported Number of Times Assaulted, Perpetrators and Types of Violence, Accessing Help After Violence Among Women in the SuperMIX Cohort, 2008-2019

Women self-reporting assault	No. (%) (N = 345)
**No. of times assaulted**	
No. of times assaulted at baseline, median (IQR)	7 (4-25)
Missing, No./total No. (%)	207/297 (70)
No. of times assaulted at follow-up, median (IQR)	3 (1-11)
Missing, No./total No. (%)	0/178 (0)
No. of times assaulted in lifetime, median (IQR)	5 (2-17)
Missing, No./total No. (%)	101/345 (29)
Perpetrator of assault or violence^a^	
Stranger	149 (43)
Drug dealer or other person who uses drugs	102 (30)
Police or security guard	46 (13)
Partner^b^	181 (53)
Other family member or friend	133 (39)
Sex trade worker or client	17 (5)
Sexual partner	24 (7)
Ex-partner	89 (26)
Other	54 (16)
Types of assault or violence^a^	
Beating or strangling	307 (89)
Attacked or threatened with weapons	158 (46)
Attacked or threated without weapons	129 (37)
Robbery, general drug violence, other	141 (41)
Types of medical care accessed^a^	
Ever accessed help	130 (38)
Accessed help during study period	77 (22)
General practitioner and primary health care	58 (17)
Emergency department	77 (22)
Other	12 (4)
Sought counseling	96 (28)

^a^ Categories are not mutually exclusive and participants could record multiple responses.

^b^ Partner includes husband, wife, partner, de facto partner, boyfriend, or girlfriend.

Of the 304 women who completed a follow-up survey, 178 (59%) self-reported at least 1 assault, of whom 176 had a valid health care–seeking response and were included in the subanalysis (ie, 2 women were excluded due to missing outcome data and 409 surveys were included) (Table 3). Of the 409 follow-up surveys reporting assault, seeking health care was reported in approximately one-third (128 [31%]). Characteristics were similar when surveys were stratified by health care seeking (Table 3). For the regression model, 14 (of 178) women were excluded due to missing covariate data, leaving 372 surveys from 164 women (mean [SD], 2.3 [1.5] surveys/woman; range, 1-9). Women reporting physical assault had an AOR of 2.28 for seeking health care (95% CI, 0.89-5.81; *P* = .08), while women reporting sexual assault had 4 times the odds (AOR, 4.13 [95% CI, 2.22-7.69]; *P* < .001) (Table 3). Women who reported having children removed from their care had higher odds of seeking health care, as did those who recently visited a GP.

**Table 3.** Sociodemographic and Drug Use Characteristics and Associations With Seeking Medical Care After Experiencing Assault or Violence Among 176 Women in the SuperMIX Cohort, 2008-2019

Characteristic	No. of surveys (%)			OR (95% CI)	*P* value	AOR (95% CI)^a^	*P* value
	Total (N = 409)	Did not seek medical care (n = 281 [69%])	Sought medical care (n = 128 [31%])				
Follow-up							
No. of interviews, median (IQR)	2 (1-3)	2 (1-3)	2 (1-3)	NA	NA	NA	NA
Assault							
Physical assault	363 (89)	246 (88)	117 (91)	1.47 (0.69-3.15)	.32	2.28 (0.89-5.81)	.08
Sexual assault	55 (13)	25 (9)	30 (23)	3.14 (1.85-5.32)	<.001	4.13 (2.22-7.69)	<.001
Missing	7 (2)	4 (1)	3 (2)	NA	NA	NA	NA
Reported any assault at baseline	323 (79)	219 (78)	104 (81)	NA	NA	NA	NA
Missing	26 (6)	16 (6)	10 (8)	NA	NA	NA	NA
No. of times assaulted, median (IQR)	2 (1-4)	2 (1-4)	2 (1-4)	NA	NA	NA	NA
Missing	51 (13)	36 (13)	15 (12)	NA	NA	NA	NA
Participant characteristics							
Age at interview, mean (SD), y	32.1 (5.6)	32.2 (5.8)	31.8 (4.9)	NA	NA	NA	NA
Aboriginal and/or Torres Strait Islander	50 (12)	34 (12)	16 (13)	NA	NA	NA	NA
Missing	5 (1)	4 (1)	1 (1)	NA	NA	NA	NA
Housing stability							
Stable	339 (83)	235 (84)	104 (81)	1 [Reference]	NA	1 [Reference]	NA
Unstable	38 (9)	24 (9)	14 (11)	1.23 (0.58-2.60)	.58	0.81 (0.34-1.95)	.65
Sleeping rough or unhoused	32 (8)	22 (8)	10 (8)	0.79 (0.31-2.01)	.62	0.82 (0.28-2.36)	.71
Weekly income							
No income	0	0	0	NA	NA	NA	NA
$1-599	214 (52)	145 (52)	69 (54)	1 [Reference]	NA	1 [Reference]	NA
$600-$2500	190 (47)	135 (48)	55 (43)	0.80 (0.50-1.28)	.36	0.77 (0.45-1.34)	.36
Missing	5 (1)	1 (0.4)	4 (3)	NA	NA	NA	NA
No paid work	361 (88)	250 (89)	111 (87)	0.77 (0.41-1.43)	.40	0.53 (0.27-1.07)	.08
Paid sex work	33 (8)	22 (8)	11 (9)	NA	NA	NA	NA
Missing	242 (59)	167 (60)	75 (59)	NA	NA	NA	NA
Sexual orientation							
Heterosexual	207 (51)	144 (51)	63 (49)	NA	NA	NA	NA
LGBTQIA+	84 (21)	56 (20)	28 (22)	NA	NA	NA	NA
Missing	118 (29)	81 (29)	37 (29)	NA	NA	NA	NA
Living arrangements							
Spouse or partner, or ex-spouse or ex-partner	72 (18)	51 (18)	21 (16)	1 [Reference]	NA	1 [Reference]	NA
Spouse or partner and children	35 (9)	24 (9)	11 (9)	1.06 (0.42-2.64)	.90	1.30 (0.49-3.47)	.60
Children, no spouse or partner	74 (18)	48 (17)	26 (20)	1.19 (0.60-2.37)	.62	1.37 (0.63-3.01)	.43
Other living arrangement	227 (56)	157 (56)	70 (55)	0.96 (0.54-1.70)	.89	0.87 (0.46-1.65)	.66
Missing	1 (0.2)	1 (0.4)	0	NA	NA	NA	NA
Children removed (since last interview)							
No	97 (24)	67 (24)	30 (23)	NA	NA	NA	NA
Yes	38 (9)	26 (9)	12 (9)	NA	NA	NA	NA
No children in care	23 (6)	17 (6)	6 (5)	NA	NA	NA	NA
Missing	251 (61)	171 (61)	80 (63)	NA	NA	NA	NA
History of child removal							
No	78 (19)	60 (21)	18 (14)	1 [Reference]	NA	1 [Reference]	NA
Yes	146 (36)	97 (35)	49 (38)	1.68 (0.87-3.24)	.12	2.34 (1.13-4.84)	.02
No children in care	184 (45)	123 (44)	61 (48)	1.58 (0.87-2.86)	.13	1.82 (0.92-3.59)	.09
Missing	1 (0.2)	1 (0.4)	0	NA	NA	NA	NA
Daily injecting drugs in past week							
Not injecting drugs in this time frame	107 (26)	71 (25)	36 (28)	1 [Reference] (combined)^b^	NA	1 [Reference] (combined)^b^	NA
Less than daily	173 (42)	114 (41)	59 (46)	1 [Reference] (combined)^b^	NA	1 [Reference] (combined)^b^	NA
Daily or more	129 (32)	96 (34)	33 (26)	0.71 (0.43-1.17)	.18	0.76 (0.41-1.40)	.38
Percentage of the time injected drugs alone							
0%-49% of the time (or did not use)	280 (69)	196 (70)	84 (66)	1 [Reference]	NA	1 [Reference]	NA
50%-100% of the time	127 (31)	83 (30)	44 (34)	1.46 (0.92-2.32)	.11	1.43 (0.82-2.49)	.20
Missing	2 (1)	2 (1)	0	NA	NA	NA	NA
Past 6 mo opioid overdose	37 (9)	26 (9)	11 (9)	NA	NA	NA	NA
Missing	171 (42)	118 (42)	53 (41)	NA	NA	NA	NA
Past 6 mo any overdose	39 (10)	27 (10)	12 (9)	NA	NA	NA	NA
Missing	171 (42)	118 (42)	53 (41)	NA	NA	NA	NA
Recent incarceration	110 (27)	73 (26)	37 (29)	1.28 (0.82-2.00)	.27	1.23 (0.75-2.02)	.42
Missing	4 (1)	3 (1)	1 (1)	NA	NA	NA	NA
Prescribed OAT	228 (56)	152 (54)	76 (59)	1.25 (0.75-2.09)	.40	1.00 (0.54-1.86)	>.99
Recent GP non-OAT visit^c^	308 (75)	203 (72)	105 (82)	1.78 (1.06-3.01)	.03	1.79 (1.03-3.12)	.04

Abbreviations: AOR, adjusted odds ratio; GP, general practitioner; LGBTQIA+, lesbian, gay, bisexual, transgender, queer, intersex, asexual, and other sexual and gender minority; NA, not applicable; OR, odds ratio; OAT, opiate agonist therapy.

^a^ Adjusted model was a complete-case analysis and included 372 interviews from 164 women (range of interviews, 1-9; mean [SD] of interviews, 2.3 [1.5]).

^b^ Two categories have been combined and the variable in the model is a binary variable.

^c^ Recent visit indicates since last interview.

### Experiences of Accessing Services for Violence

Eight (of 10) women described experiencing violence from past or current partners, several reported multiple violent relationships, and some reported consequent hospitalization. Most women described difficulty accessing health and social services after violence, and some chose not to seek support. Although GPs were recognized as potential first points of help, most women lacked a regular GP because it was difficult to find someone they trusted and they often felt stigmatized due to their drug use.

… everybody should have a relationship with their GP, but unfortunately, …there’s some that won’t listen to you. They say, “Oh, you’re a drug user and we don’t want to know you” …There’s a lot of doctors that look down at you as soon as they know that you’ve been a drug user before. It wouldn’t matter how many years ago, you could’ve stopped [using drugs]. (Mira, in her 50s)

The prospect of having to tell or retell their story, including disclosing their history of injecting drug use, prevented some women from accessing family violence services.

I get anxious about going to the doctor… like, to keep telling everything over and over and it’s like really heavy stuff… That’s really a big issue for me… all the things that add up to my medical history, having to tell every single new doctor a summary of it all and everything… I can look back and see now that there was instances [sic] where I’ve had anxiety… but… I didn’t look at it like that until after the domestic violence situation, and it’s definitely more so since then. (Sally, in her 40s)

Some women who experienced violence said that fear their children would be removed because of their drug use had prevented them from seeking help, accessing services, or reporting abuse to police.

I was too scared—child protection and everything… We need somebody that doesn’t go straight to child protection… if I had somebody that I could talk to and work with, I believe I would’ve opened up and I could have got [sic] help that way, but I was too scared… because I knew I’d get into trouble… (Sara, in her 40s)

Other barriers included a lack of access to transport to attend in person and lacking a telephone or credit to call services. Eve, for example, had been in abusive relationships but never accessed services for support, despite believing she should have. She reflected: *“*I’m not sure why. Probably different reasons for different partners at different times [but] one would maybe be transport.” Eve described these practical barriers:

I’ve noticed lately that you have to ring for an appointment before you go to see [family violence services]. And I don’t think that’s fair because some people haven’t got phones. And if they do have phones, they don’t have credit. [One service] made it so that you could ring a certain line and it wouldn’t cost anything to ring them, but still you’ve got to get out and find a pay phone… [and] some of the phones don’t even do the free calls or anything. (Eve, in her 50s)

Interviews elucidated the importance of trusted relationships with health care practitioners for facilitating referrals to family violence services or supporting women to leave abusive relationships. Two women described positive experiences in which caring, trusted clinicians played a decisive role. Jane recalled a maternal child health nurse who recognized signs of abuse and prompted a referral, enabling her to seek refuge:

She sensed that there was help needed. I was, yeah, going to [regional hospital] for my maternal check-ups with my third baby… then when I reconnected with the [hospital name], she’d seen my name pop up and she came into one of the visits and she said, “I want to touch base. How’s things going?” …I was just lucky that she’d stuck with me from the third child to my fourth child. …They made me feel so safe and everything because they actually got me out of [town] and put me through the refuge system… Got me housing… Fully supported me with the 4 kids. It was fantastic. (Jane, in her 40s)

Mira said her opioid agonist treatment prescriber had asked about her relationship out of concern for her safety, and that this had encouraged her to leave:

Even when my boyfriend beat me up or anything like that, [my prescriber], he’d say to me, “Well, do you want me to make a complaint about your injuries or anything like that?” because I’d got black eyes and broken ribs… the first one, he just said, “Right. I can’t handle this anymore because you’re getting too much black eyes” and when my arm was broken, I just got rid of him… I stood up for myself then because I had more encouragement. (Mira, in her 50s)

## Discussion

In a cohort of Australian women who inject drugs, most experienced violence: 82% experienced at least 1 assault in their lifetime, and well over one-third (38%) experienced at least 1 sexual assault; these rates are roughly double those reported among women in the general population for violence (39%) and sexual violence (22%).^22^ In addition, they exceed rates of violence reported among Australian women from culturally and linguistically diverse backgrounds (33%), women with a disability (64%), and LGBTIQA+ people (65%), populations formally recognized for their increased risk and burden of violence.^13^ Only one-third of women who experienced violence sought health care after assault despite almost 1 in 4 presenting to the ED with suspected assault and 1 in 5 hospitalized during the study period. Sexual assault and child removal were associated with higher likelihood of seeking health care. Qualitative findings identified several barriers to accessing care after violence, including drug-related stigma, repeated disclosure, lack of a trusted GP, and practical barriers such as no telephone or transport. These findings highlight that women who inject drugs should be recognized in Australia’s National Plan to End Violence Against Women and Children and for targeted, responsive support to address the substantial harms they have experienced.^13^ Without recognition and targeted support for women at highest risk, efforts to reduce violence against women are unlikely to succeed.

The violence experienced by women who inject drugs is likely shaped by intersecting structural and systemic factors,^9,10^—including criminalization of drug use, lack of safe housing, inadequate harm reduction and treatment coverage, stigma and discrimination, and entrenched poverty—that compound social and economic marginalization.^23^ These factors interact with individual-level risks, which are more prevalent among women who inject drugs, including mental illness, unhoused status, sex work, and reliance on intimate partners for drugs.^24^ The combination of a high prevalence of violence and low rates of health care seeking suggest an urgent need for intersectional harm reduction and violence response services tailored to women who inject drugs. Existing low-threshold harm reduction services are often male dominated, both in Australia and globally,^25^ whereas women-only and gender-diverse spaces staffed by peer workers with lived experience have shown greater effectiveness,^26^ underscoring the need for investment in peer-led workforce development.

Although intimate partner violence was common, participants reported assault from a range of people. Most women identified partners as the perpetrators of violence, as in the Australian general population^22^; however, nearly half (43%) also reported being assaulted by a stranger, a significantly higher rate than among the general population.^22^ Many women identified other family members, friends, and drug dealers or other people who use drugs as perpetrators of violence. This finding underscores a critical gap in existing support services for women who inject drugs, which are designed primarily to respond to domestic or family violence. Furthermore, current strategies for seeking support for stranger-perpetrated violence rely on engagement with law enforcement, which many women who inject drugs distrust or fear due to experiences of criminalization, discrimination, or past trauma.^27^

Seeking health care after assault was uncommon among women. Beyond known barriers, such as fear of child protection services involvement and safety concerns,^18,28^ women also identified drug-related stigma and practical challenges, such as no telephone credit. Women who inject drugs often face misogyny and harmful stereotypes that shape interactions with health care and social services workers, even those intended to support people who use drugs.^29^ Many domestic violence and crisis services exclude people currently using drugs.^30^ Although recommendations in Australia’s *Unlocking the Prevention Potential* report call for better collaboration between alcohol and other drug services and violence services,^31^ implementation remains unclear, and the National Plan to End Violence Against Women and Children does not specify the role of these services.^13^ Integrated, low-threshold models of care are therefore needed to increase help seeking by women who inject drugs. Our findings showed that GPs emerged as key contact points, yet mistrust of GPs and fear of child removal remain major deterrants to contacting GPs.^28^ These fears are reinforced by Australian child welfare policies that disproportionately monitor parents who use drugs,^18^ framing them as unfit to parent and likely contributing to the persistent underdisclosure of violence.^32^

### Limitations

This study has some limitations. We used multiple data sources to explore violence against women with lived experience of injecting drug use, filling a significant research gap. Although our findings offer valuable insight, the study was descriptive and did not establish causal relationships between participant characteristics and health care seeking after assault.^33^ Varying recall periods made determining sequences of events difficult, and self-reported data are subject to recall and social desirability bias. Clinical records may underreport or misclassify assault, leading to underestimation. We did not apply weighting for nonresponse or loss to follow-up, which may introduce selection bias, although linkage with administrative data to capture outcomes strengthens the analysis. The qualitative component, while not focused on violence, provided valuable contextual depth. This study lays a strong foundation for research on causal pathways for violence and help seeking among women who inject drugs.

## Conclusions

In this mixed-methods cohort study among women who inject drugs in Australia, violence perpetrated against women occurred at double the observed prevalence in the general population. Over the study period, 1 in 5 women were admitted to hospital due to an assault, attesting to its severity. However, seeking health care after an assault was uncommon, with women citing stigma, fears of child removal, and structural barriers as reasons for not seeking health care. A multifaceted response to violence against women that incorporates women-centric and peer-led harm reduction services and crisis responses within alcohol and other drug settings is needed because violence and substance use often intersect and cannot be addressed in isolation. Finally, the high risk and burden of violence faced by women who inject drugs should be formally recognized in the quest to end violence against women and children in Australia.

## Notes

Supplement 1. **eTable 1.** Survey Items and Response Options Used to Derive Experiences of Assault and Estimate Violence Against Women Who Inject Drugs **eTable 2.** Data Used to Derive Outcome Variables Estimating Violence Against Women Who Inject Drugs **eFigure.** Participant Flow Chart

Supplement 2.
Data Sharing Statement
