# Supplementary material for: Violence Against Women Who Inject Drugs
Source: JAMA Netw Open. 2026 Mar 17;9(3):e262096. doi: 10.1001/jamanetworkopen.2026.2096 (PMC13446803; doi:10.1001/jamanetworkopen.2026.2096)
Supplement: Supplementary file 1 [file jamanetwopen-e262096-s001.pdf]

Supementary Online Content

Colledge-Frisby S, Walker S, Wilkinson AL, et al. Violence against women who inject drugs. *JAMA Netw Open.* 2026;9(3):e262096. doi:10.1001/jamanetworkopen.2026.2096

eTable 1. Survey Items and Response Options Used to Derive Experiences of Assault and Estimate Violence Against Women Who Inject Drugs

eTable 2. Data Used to Derive Outcome Variables Estimating Violence Against Women Who Inject Drugs

eFigure. Participant Flow Chart

This supplementary material has been provided by the authors to give readers additional information about their work.

© 2026 Colledge-Frisby S et al. *JAMA Network Open.*

eTable 1. Survey Items and Response Options Used to Derive Experiences of Assault and Estimate Violence Against Women Who Inject Drugs

Question	Response options***
Have you been attacked, assaulted (including sexual assault) or suffered any kind of violence?	Yes<br>No
How many times have you been assaulted?*	[Number of times indicated]
Who has attacked you? [multiple responses allowed]*	Stranger<br>Dealer<br>Police<br>Husband/wife/partner/de facto<br>Boyfriend/girlfriend<br>Sex trade worker/mug<br>Friend<br>Regular sex partner<br>Casual sex partner<br>Security guard/Protective services officer<br>Other drug user<br>Other family member<br>Ex-partner<br>Other
What types of attacks on you have occurred?*	Beating/punched and kicked<br>Sexual assault/rape<br>Attacked with weapons (e.g. club, knife, belt, bottle)<br>Strangled/choked<br>Attacked or threatened (generally)<br>Attacked or threatened with a gun<br>Robbery (stood over/rolled for drugs or money)<br>General drug violence<br>Other
Did you seek medical attention (health care) after any of these attacks?*	No<br>Some of the time<br>Most of the time<br>All of the time
Where did you go?**	General practitioner<br>IDU primary health centre<br>Emergency department<br>Other

* These questions followed branching logic, such that only individuals who responded yes to being assaulted were asked these questions.
** As above, individuals who responded yes to whether they sought medical attention were asked this follow-up question.
*** Do not know and refused to answer were response options for all questions.

© 2026 Colledge-Frisby S et al. *JAMA Network Open.*

eTable 2. Data Used to Derive Outcome Variables Estimating Violence Against Women Who Inject Drugs

**Outcome**	**Self-report data**	**Hospital data**	**ED data**	**Ambulance data**	**Mortality data**
Any assault	Self-reported assault at baseline or follow-up survey.	Admissions with diagnoses of ICD-codes X85-Y05, Y07-09.	Presentations with diagnoses of ICD-codes X85-Y05, Y07-09, or clinician-assessed assault of external injury presentation.	Callouts with event coding 315, 316, 4A1A, 4A1S, 4A2A, 4A2S, 4B1A, 4B1S, 4B2A, 4B2S, 4B3A, 4B3S, 4D1A, 4D1S, 4D2A, 4D2S, 4D3A, 4D3S, 4D4A, 4D4S.	Cause of death diagnoses of ICD-codes X85-Y05, Y07-09.
Sexual assault	Self-reported sexual assault at baseline or follow-up survey.	Admissions with diagnoses of ICD-code Y05.	Presentations with diagnoses of ICD-code Y05, or clinician-assessed sexual assault of external injury presentation.	-	Cause of death diagnoses of ICD-code Y05.
Physical assault	Self-reported physical assault at baseline or follow-up survey.	Admissions with diagnoses of ICD-codes X85-Y04, Y07-09.	Presentations with diagnoses of ICD-codes X85-Y04, Y07-09.	-	Cause of death diagnoses of ICD-codes X85-Y05, Y07-09.
Accessed help for assault	Self-reported accessing medical attention/health care after assault in follow-up surveys only.	-	-	-	-

ICD-10, International Classification of Diseases, 10th edition
ED, emergency department

© 2026 Colledge-Frisby S et al. *JAMA Network Open.*

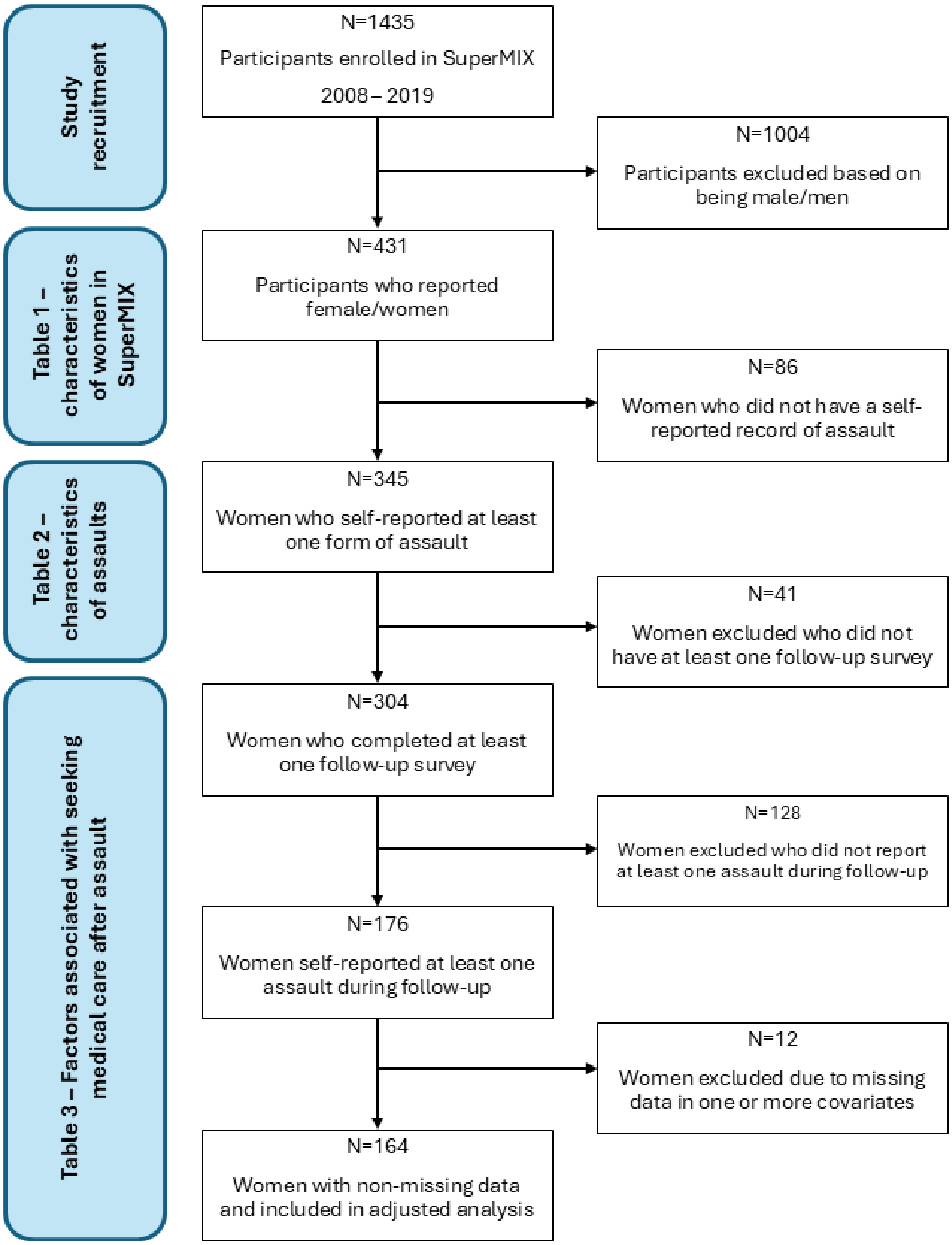

eFigure. Participant Flow Chart

© 2026 Colledge-Frisby S et al. *JAMA Network Open.*
