# Supplementary material for: Violence Against Women Who Inject Drugs
Source: JAMA Netw Open. 2026 Mar 17;9(3):e262096. doi: 10.1001/jamanetworkopen.2026.2096 (PMC13446803; doi:10.1001/jamanetworkopen.2026.2096)
Supplement: Supplementary file 2 [file jamanetwopen-e262096-s002.pdf]

Data Sharing Statement

Colledge-Frisby. Violence Against Women Who Inject Drugs. *JAMA Netw Open*. Published March 17, 2026. doi:10.1001/jamanetworkopen.2026.2096

Data
Data available: No

Additional Information
Explanation for why data not available: The data supporting this study are not publicly available due to privacy and ethical constraints. However, they may be made available upon reasonable request to the study's principal investigator, subject to institutional and ethical approvals.
